# Effect of apolipoprotein C3 genetic polymorphisms on serum lipid levels and the risk of intracerebral hemorrhage

**DOI:** 10.1186/s12944-015-0047-9

**Published:** 2015-05-22

**Authors:** Yan Jiang, Junpeng Ma, Hao Li, Yi Liu, Chao You

**Affiliations:** Department of Neurosurgery, West China Hospital, Sichuan University, 37 Guoxuexiang Street, Chengdu, 610041 China

**Keywords:** Intracerebral hemorrhage, Lipid, Apolipoprotein C3, Gene polymorphism

## Abstract

**Background:**

Serum lipid levels are associated with the risk of intracerebral hemorrhage (ICH). Genetic variants in the apolipoprotein C3 (*APOC3*) gene were associated with plasma triglyceride (TG) and very-low-density lipoprotein (VLDL) levels. The aim of this study was to evaluate the effect of two genetic variants (1100 C/T and 3238 C/G) of *APOC3* on serum lipid levels and risk of ICH.

**Methods:**

A prospective hospital-based case–control design and logistic regression analysis were utilized. We enrolled 150 ICH patients and 150 age- and gender-matched controls. The *APOC3* gene polymorphisms were determined by polymerase chain reaction-restriction fragment length polymorphism (PCR-RFLP).

**Results:**

ICH patients had a significantly higher frequency of *APOC3* 3238 GG genotype [odds ratio (OR) =2.97, 95 % confidence interval (CI) = 1.20, 7.38; *P* = 0.02] and *APOC3* 3238 G allele (OR =1.53, 95 % CI = 1.03, 2.27; *P* = 0.04) than controls. The *APOC3* 3238 G allele was significantly associated with increasing plasma TG levels and VLDL levels both in ICH cases (*P* = 0.01) and controls (*P* = 0.02). No association was found between *APOC3* 1100 C/T polymorphisms and ICH.

**Conclusion:**

To the best of our knowledge, this is the first report in the literature that the *APOC3* 3238 GG genotype and G allele might contribute to an increased risk of ICH as a result of its effect on serum lipid levels.

## Introduction

Intracerebral hemorrhage (ICH) occurs at an annual incidence rate of 15 to 19 per 100,000 [1]. ICH accounts for about 15 % of all strokes and is associated with 3-month mortality of approximately 25 % [1, 2]. Even with state-of-the-art medical care, ICH results in death, or severe disability in more than 50 % of cases [3, 4]. ICH may occur due to hypertension, diabetes mellitus, vascular malformation, menopause, cerebral amyloid angiopathy, current cigarette smoking, trauma, coagulopathy [5–9], and serum lipid levels [10–12].

Apolipoprotein C3 (*APOC3*) is a major component of triglyceride (TG)-rich lipoproteins, and a minor component of high-density lipoprotein (HDL) [13]. *APOC3* gene, located in the chromosome 11q23, was involved in transport, and clearance of chylomicron remnants, and very-low-density lipoprotein (VLDL), and HDL from the bloodstream [14, 15]. *APOC3* encodes a 79-amino-acid glycoprotein produced mainly in the liver inhibiting the action of lipoprotein lipase and interfering with receptor-mediated lipoprotein uptake [16]. Two common single nucleotide polymorphisms (SNPs) have been identified in the APOC3 gene: 1100 C/T and 3238 C/G [17, 18]. Genetic variants in the *APOC3* gene were associated with plasma TG and VLDL levels [19, 20].

We hypothesized that common genetic variants in *APOC3* gene influenced the risk of ICH. To test this hypothesis, we performed a prospective hospital-based case–control study to evaluate the effect of two genetic variants (1100 C/T and 3238 C/G) of *APOC3* on serum lipid levels and risk of ICH.

## Materials and methods

### Study population

This is a prospective hospital-based case–control study between July 2011 and July 2013 in the Department of Neurosurgery, West China Hospital, Sichuan University, China. We enrolled 150 ICH patients and 150 age- and gender-matched controls. Eligibility for ICH patients required neuroimaging (CT or MRI) confirmation of hemorrhagic stroke. Exclusion criteria were defined as: presence of a vascular malformation, aneurysmal subarachnoid hemorrhage, hemorrhagic transformation of acute infarction, traumatic ICH, brain neoplasm, or any other suspected cause of secondary ICH. Controls were confirmed to have no medical history of ICH, Alzheimer’s disease, or pre-enrollment dementia by means of interview and review of medical records. In addition, similar to the cases the controls were all required to be born in China to native Chinese Han parents. To confirm the diagnosis, two physicians reviewed the hospital records and validated each case. Collected clinical data included age, sex, body mass index (BMI), smoking status, and medical history including hypertension, diabetes mellitus, hyperlipidemia, ischemic stroke, and previous ICH. Medications included the use of warfarin, antiplatelet therapy, and statins. All data points were collected through interviews with the patient or their families/surrogates. All parts of the study were approved by the Institutional Ethical Committee of the West China Hospital, Sichuan University, and informed consent according to the Declaration of Helsinki was obtained from all participants or their families/surrogates.

### DNA extraction and genotyping

Genomic DNA was isolated from white blood cells by the commercially available Qiagen kit (QIAGEN Inc., Valencia, CA, USA). The *APOC3* gene polymorphisms were determined by polymerase chain reaction-restriction fragment length polymorphism (PCR-RFLP). Briefly, the primers designed for *APOC3* 1100 C/T and 3238 C/G were 5′-AGA GGC CGA TCC ACC CCA CTC AGC C-3′ (forward) and 5′-GGC GGT CTT GGT GGC GTG CTT CAG G-3′ (reverse); 5′-CAT GGT TGC CTA CAG AGG AGT-3′ (forward) and 5′-TGA CCT TCC GCA CAA AGC TGT-3′ (reverse), respectively. The amplified PCR products were digested with *Sst*I (3238 C/G) and *Sac*I (1100 C/T) (New England BioLabs, Missisauga, ON). Details of PCR conditions have been described elsewhere [21]. Electrophoresis in a 2.5 % agarose gel followed by ethidium bromide staining and ultraviolet illumination allowed detection of the alleles. For quality control, two independent observers, read all genotypes without knowing about the case or control status. When replicate quality control samples were evaluated, genotypes showed 100 % concordance.

### Biochemical analysis

Serum total cholesterol (TC), triglycerides (TG), high-density lipoprotein cholesterol (HDL-C) were measured by the clinical chemistry department using commercial kits following the manufacturer instructions. Commercial kits for measuring TC, TG, HDL-C, and LDL-C were obtained from Beijing BHKT Clinical Reagent Co., Ltd. (Beijing, China). All of the serum samples were measured with a SpectraMax M2 microplate reader (Molecular Devices, USA). Serum low-density lipoprotein cholesterol (LDL-C) and very-low-density lipoprotein cholesterol (VLDL-C) were calculated using the Friedwald’s formula. The immunoturbidimetric assay (BioSino Bio-technology and Science Inc., Beijing, China) was used to quantify the plasma concentrations of ApoA1 and ApoB. All assays were conducted following the manufactures’ instruction.

### Statistical analysis

SAS version 9.1 (SAS Institute, Cary, NC) was used for all statistical tests. Data are presented as means ± standard deviation (SD) or as percentages for categorical variables. Differences between continuous variables were assessed by Student’s *t* test, while those between categorical variables were evaluated using Pearson *x*^2^ test. To eliminate confounding influences, a binary logistic-regression model was used to determine ICH risk (estimated by the odds ratio [OR] with 95 % confidence interval [95 % CI]) by adjustment for conventional risk factors, including age, sex, BMI, smoking status, history of hypertension, or diabetes, total cholesterol, HDL-C, and LDL-C. We also have adjusted for medicine use by which lipid levels are affected in this analysis. The plasma lipid levels were compared among different genotypes by ANCOVA, adjusted for gender, age, and BMI. Statistical significance was taken at nominal *P*-value < 0.05 for all comparisons.

## Results

### Characteristics of participants

General characteristics of ICH patients and controls were presented in Table 1. There were no significant differences between the ICH patients and controls in age, sex, BMI, ApoA1, and ApoB (Table 1). The proportions of smoking status (*P* = 0.03), hypertension (*P* < 0.001), diabetes (*P* = 0.001), and hyperlipidemia (*P* = 0.004) were significantly higher, and levels of TG (*P* = 0.005), TC (*P* = 0.004), LDL (*P* = 0.001) and VLDL (*P* = 0.005) were significantly higher, and levels of HDL were significantly lower (*P* = 0.02) in ICH patients than in controls (Table 1).

**Table 1:** General characteristics of ICH patients and controls

Variables	ICH	Controls	*P*
Number of subjects	150	150	
Age (years), (mean ± SD)	65.3 ± 11.2	64.8 ± 10.9	0.69
Sex (Male/Female)	89/61	92/58	0.72
BMI (Kg/m^2^)	25.7 ± 3.3	25.3 ± 3.2	0.29
Smoking status (Ever/Never)	31/119	17/133	0.03
Hypertension (Positive/Negative)	91/59	43/107	<0.001
Diabetes (Positive/Negative)	35/115	14/136	0.001
Hyperlipidemia (Positive/Negative)	43/107	22/128	0.004
TG (mg/dL)	176.3 ± 87.5	149.8 ± 75.6	0.005
TC (mg/dL)	196.8 ± 59.5	178.4 ± 51.4	0.004
HDL (mg/dL)	34.5 ± 15.6	38.9 ± 16.7	0.02
LDL (mg/dL)	127.0 ± 46.5	109.5 ± 43.7	0.001
VLDL (mg/dL)	35.3 ± 17.5	30.0 ± 15.1	0.005
ApoA1 (mg/dL)	124.1 ± 21.5	122.7 ± 21.8	0.58
ApoB (mg/dL)	89.3 ± 19.4	88.5 ± 19.2	0.72

*ICH* intracerebral hemorrhage, *BMI* body mass index, *TG* triglyceride, *TC* total cholesterol, *HDL* high-density lipoprotein, *LDL* low-density lipoprotein, *VLDL* very-low-density lipoprotein, *Apo* apolipoprotein

### APOC3 3238 C/G polymorphisms, serum lipid levels, and ICH

ICH patients had a significantly higher frequency of *APOC3* 3238 GG genotype (OR =2.97, 95 % CI = 1.20, 7.38; *P* = 0.02) and *APOC3* 3238 G allele (OR =1.53, 95 % CI = 1.03, 2.27; *P* = 0.04) than controls (Table 2). The *APOC3* 3238 G allele was significantly associated with increasing plasma TG levels and VLDL levels both in ICH cases (*P* = 0.01) and controls (*P* = 0.02) (Table 3).

**Table 2:** Genotype and allele frequencies of *APOC3* gene polymorphisms among ICH cases and healthy controls

Genotypes	ICH (%)	Controls (%)	OR (95%CI)	*P*
1100 CC	58(38.7)	63(42.0)	1.00(Reference)	
1100 CT	65(43.3)	53(35.3)	1.33(0.80,2.22)	0.27
1100 TT	27(18.0)	34(22.7)	0.86(0.47,1.60)	0.64
1100 C allele frequency	181(60.3)	179(59.7)	1.00(Reference)	
1100 T allele frequency	119(39.7)	121(40.3)	0.97(0.70,1.35)	0.87
3238 CC	95(63.3)	104(69.3)	1.00(Reference)	
3238 CG	36(24.0)	39(26.0)	1.01(0.59,1.72)	0.97
3238 GG	19(12.7)	7(4.7)	2.97(1.20,7.38)	0.02
3238 C allele frequency	226(75.3)	247(82.3)	1.00(Reference)	
3238 G allele frequency	74(24.7)	53(17.7)	1.53(1.03,2.27)	0.04

Adjustment for conventional risk factors, including age, sex, BMI, smoking status, history of hypertension, or diabetes, total cholesterol, HDL-C, LDL-C, and medicine use by which lipid levels are affected

*ICH* intracerebral hemorrhage

**Table 3:** Lipid profiles of ICH cases and controls according to *APOC3* 3238 C/G polymorphisms

	ICH			*P* value	Controls			*P* value
	CC	CG	GG		CC	CG	GG	
TG (mg/dL)	159.7 ± 85.3	196.3 ± 92.1	236.7 ± 103.4	0.01	131.5 ± 68.7	173.4 ± 79.8	219.5 ± 89.6	0.02
TC (mg/dL)	192.1 ± 59.1	208.5 ± 60.8	198.8 ± 59.8	0.58	174.6 ± 51.0	191.5 ± 58.7	161.8 ± 44.9	0.83
HDL (mg/dL)	33.7 ± 17.6	38.2 ± 18.3	31.5 ± 9.8	0.74	35.8 ± 15.1	42.4 ± 19.6	35.4 ± 14.8	0.54
LDL (mg/dL)	126.5 ± 45.7	131.0 ± 48.2	120.1 ± 41.6	0.63	110.5 ± 38.1	114.4 ± 38.5	82.5 ± 33.8	0.41
VLDL (mg/dL)	31.9 ± 17.1	39.3 ± 18.4	47.3 ± 20.7	0.01	26.3 ± 13.7	34.7 ± 16.0	43.9 ± 17.9	0.02
ApoA1 (mg/dL)	122.5 ± 22.9	127.3 ± 24.5	126.1 ± 23.4	0.26	119.3 ± 20.6	132.1 ± 26.7	120.6 ± 21.5	0.37
ApoB (mg/dL)	87.2 ± 18.7	96.2 ± 21.6	86.7 ± 18.1	0.46	85.1 ± 17.7	98.0 ± 22.4	86.1 ± 18.2	0.51

*P* values were calculated by ANCOVA, adjusted for age, sex, and BMI

### APOC3 1100 C/T polymorphisms, serum lipid levels, and ICH

No association was found between *APOC3* 1100 C/T polymorphisms and ICH (Table 2).

## Discussion

In this study, we evaluated the effect of two genetic variants (1100 C/T and 3238 C/G) of *APOC3* on serum lipid levels and risk of ICH in a Chinese population. This prospective hospital-based case–control study revealed that the *APOC3* 3238 GG genotype and G allele might contribute to an increased risk of ICH as a result of its effect on serum lipid levels. No association was found between *APOC3* 1100 C/T polymorphisms and ICH. To the best of our knowledge, this is the first report in the literature that evaluated the effect of two genetic variants (1100 C/T and 3238 C/G) of *APOC3* on serum lipid levels and risk of ICH.

The *APOC3* gene polymorphisms were also associated with many other diseases. A nested case–control study demonstrated a diet-gene interaction between *APOC3* rs5128 polymorphism and the western dietary patterns in relation to metabolic syndrome risk [22]. Two other case–control study also suggested that two genetic variants (−482 C/T and −455 T/C) of *APOC3* were associated with the metabolic syndrome [23, 24]. The GENOCOR study identified −482 C > T of *APOC3* as an additive biomarker for ischemic heart disease in an Italian cohort of ischemic patients [25]. A systematic review of 20 studies comprising 15,591 participants found that *APOC3* Sst I and T-455C polymorphisms might be associated with coronary heart disease risk [17]. A case–control study suggested that the *APOC3* 3238 G allele might contribute to an increased risk of coronary artery disease as a result of its effect on TG and VLDL-C metabolism [26]. A case–control study found that the minor alleles of *APOC3* −455 T/C polymorphisms were closely associated with acute coronary syndrome [27]. A prospective case–control study suggested that *APOC3* (−455 T > C) genetic variation was involved in the susceptibility to developing nonalcoholic fatty liver disease, insulin resistance, hypertension, hypertriglyceridemia, and low HDL in the Southern Chinese Han population [28]. Another case–control study found that the polymorphisms −482 C/T and −455 T/C in *APOC3* were associated with nonalcoholic fatty liver disease and insulin resistance [29].

Although our study suggested that the *APOC3* GG genotype and G allele might contribute to an increased risk of ICH as a result of its effect on serum lipid levels, the clear mechanism of this association is unclear. The Bogalusa Heart Study found that *APOC3* 3238 C/G polymorphisms were associated with higher serum triglyceride levels [30]. A recent prospective case–control study also found that the *APOC3* 3238 G allele might contribute to an increased risk of coronary artery disease as a result of its effect on TG and VLDL-C metabolism [26]. Serum lipid levels may be associated with the risk of ICH [10–12]. We also observed that the proportions of smoking status, hypertension, diabetes, and hyperlipidemia were significantly higher, and levels of TG, TC, LDL, and VLDL were significantly higher, and levels of HDL were significantly lower in ICH patients than in controls.

Some shortcomings of this study should be mentioned. First of all, this study is limited by its size and lack of replication. Further large scale research on the role of *APOC3* in ICH and replication of our results is necessary. Second, although we have adjusted for medicine use by which lipid levels are affected in this analysis, no information could be received on the baseline lipid levels of these patients. Third, this study only considers a Chinese population that may limit the application of these findings to other ethnic populations. Fourth, ICH is induced by multiple genes and environmental factors, which were not explored in the present study. Finally, potential selection bias might have been present, because this is a hospital based case control study and the subjects may not be representative of the general population.

## Conclusion

To the best of our knowledge, this is the first report in the literature that the *APOC3* 3238 GG genotype and G allele might contribute to an increased risk of ICH as a result of its effect on serum lipid levels. We found that ICH patients had a significantly higher frequency of *APOC3* 3238 GG genotype and *APOC3* 3238 G allele than controls. The *APOC3* 3238 G allele was significantly associated with increasing plasma TG levels and VLDL levels both in ICH cases and controls. No association was found between *APOC3* 1100 C/T polymorphisms and ICH. Additional large scale studies are needed to confirm this finding.
